# Supplementary material for: Transcriptomic Insights into Mechanisms of Early Seed Maturation in the Garden Pea (Pisum sativum L.)
Source: Cells. 2020 Mar 23;9(3):779. doi: 10.3390/cells9030779 (PMC7140803; doi:10.3390/cells9030779)
Supplement: Supplementary file 1 [file cells-09-00779-s001.zip › Supplementary materials/FileS4.zip!/FileS3/TFs - Results/Summary Sheets - Modules/Group Overexpression Spots/Report.pdf]

Group Overexpression Spots

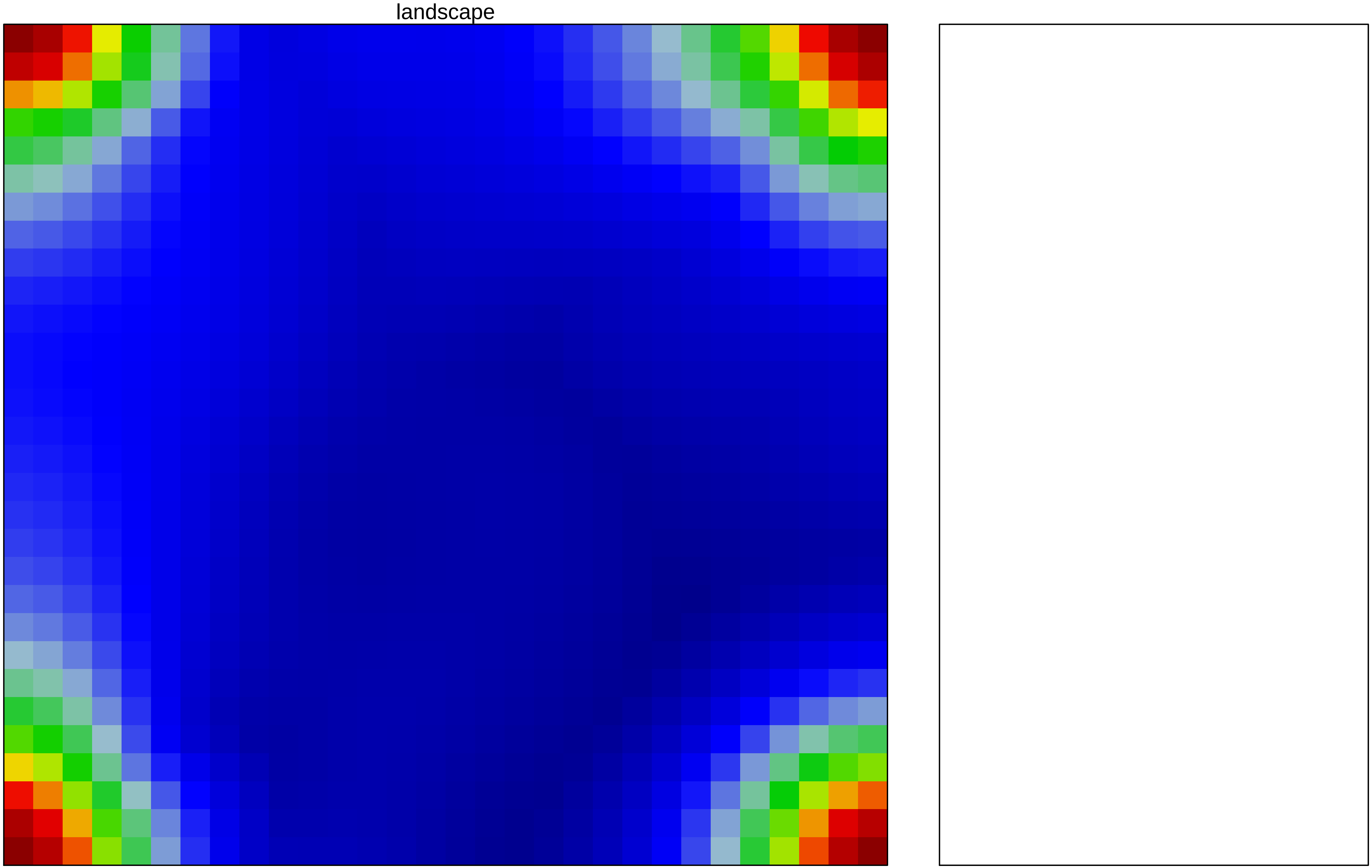

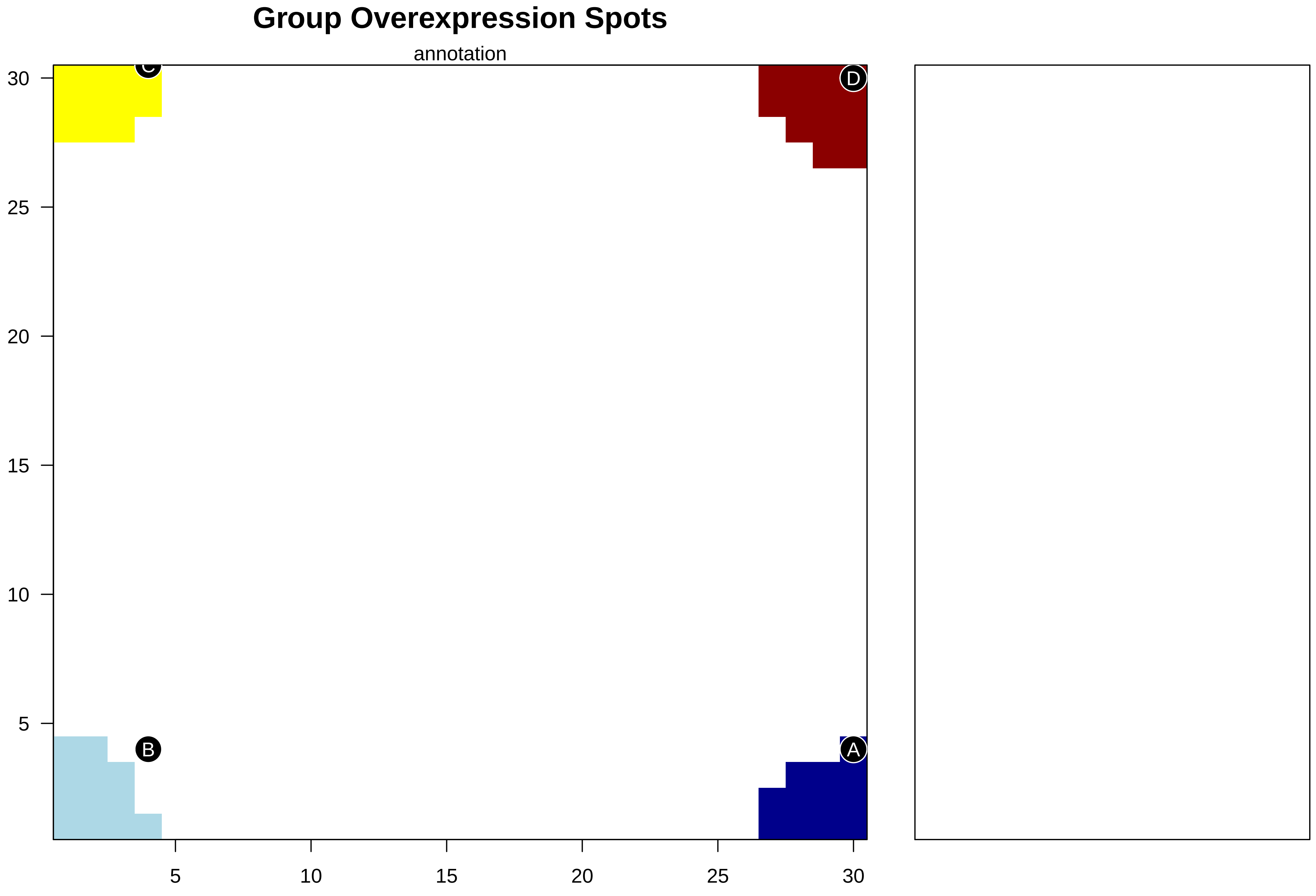
Group Overexpression Spots
annotation
A
B
C
D
5
10
15
20
25
30

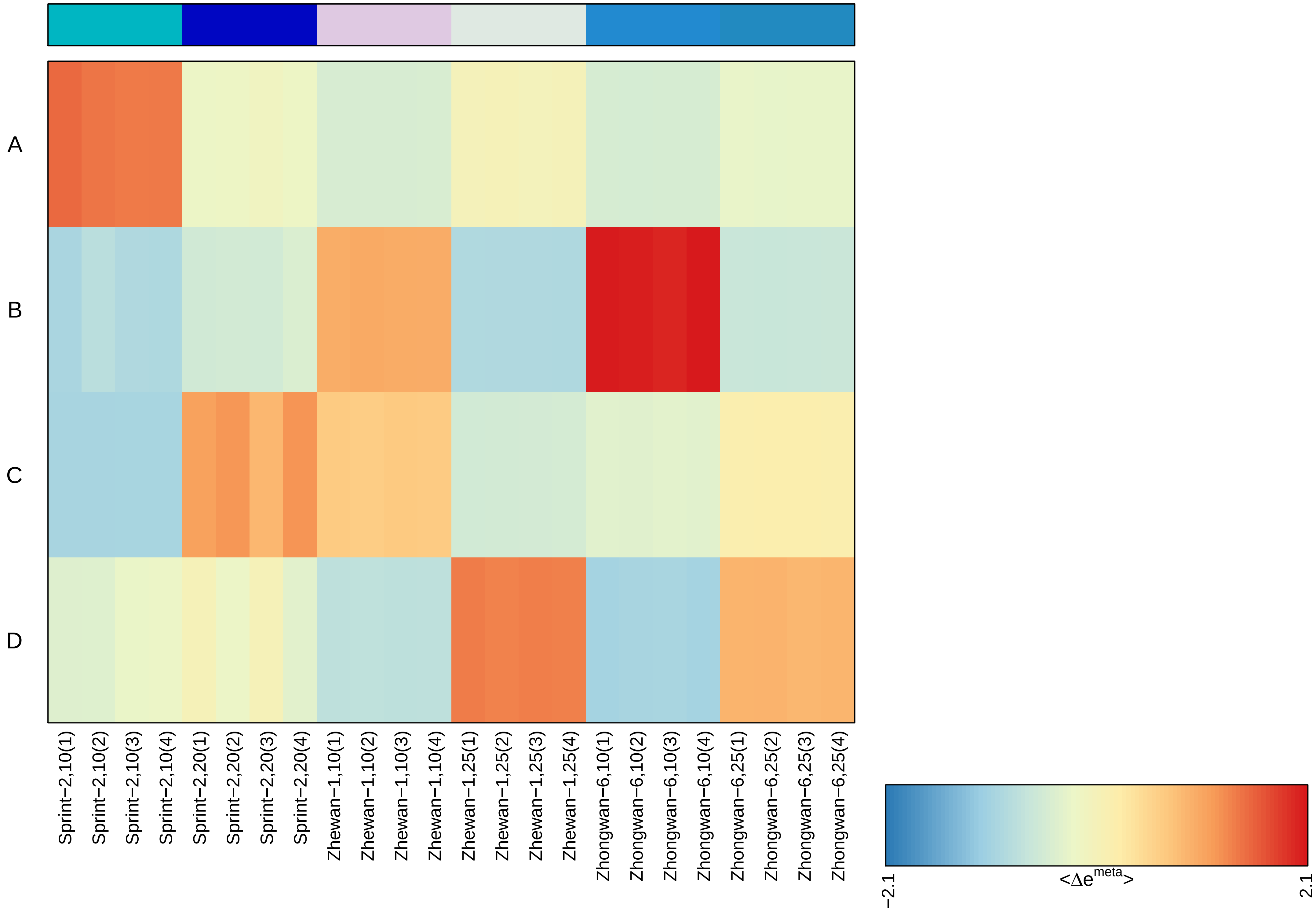

A
B
C
D
Sprint−2,10(1)
Sprint−2,10(2)
Sprint−2,10(3)
Sprint−2,10(4)
Sprint−2,20(1)
Sprint−2,20(2)
Sprint−2,20(3)
Sprint−2,20(4)
Zhewan−1,10(1)
Zhewan−1,10(2)
Zhewan−1,10(3)
Zhewan−1,10(4)
Zhewan−1,25(1)
Zhewan−1,25(2)
Zhewan−1,25(3)
Zhewan−1,25(4)
Zhongwan−6,10(1)
Zhongwan−6,10(2)
Zhongwan−6,10(3)
Zhongwan−6,10(4)
Zhongwan−6,25(1)
Zhongwan−6,25(2)
Zhongwan−6,25(3)
Zhongwan−6,25(4)
−2.1
<Δe^meta>
2.1

Group Overexpression Sp

Spot Summary: A

metagenes = 12
genes = 92

<r> metagenes = 1
<r> genes = 0.73
beta: r2= 3.14 / log p= −Inf

samples with spot = 4 (16.7 %)
Sprint−2(10) : 4 (100 %)

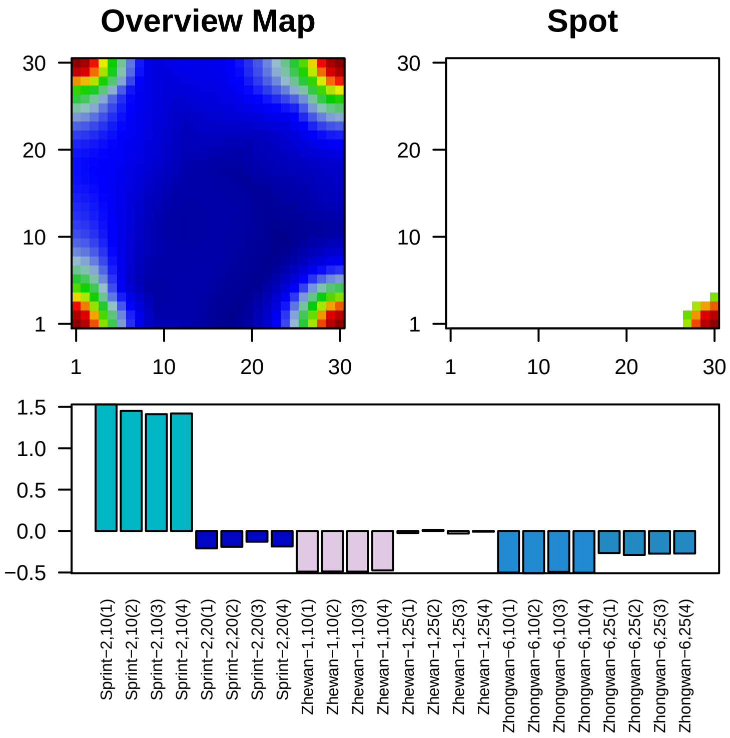

Spot Genelist

Rank	ID	max e	min e	r	Description / Symbol
1	TRINITY_DN5	8.61	−1.68	0.96	
2	TRINITY_DN2	7.6	−8.44	0.83	
3	TRINITY_DN1	7.51	−1.97	0.98	
4	TRINITY_DN1	4.85	−1.19	0.96	
5	TRINITY_DN1	4.67	−2.15	0.92	
6	TRINITY_DN6	4.43	−1.66	0.85	
7	TRINITY_DN9	3.91	−0.84	0.96	
8	TRINITY_DN7	3.84	−2.46	0.47	
9	TRINITY_DN1	3.43	−1.66	0.82	
10	TRINITY_DN1	3.36	−2.28	0.87	
11	TRINITY_DN6	3.29	−1.71	0.92	
12	TRINITY_DN1	3.27	−0.38	0.79	
13	TRINITY_DN7	3	−0.92	0.92	
14	TRINITY_DN2	2.98	−3.37	0.68	
15	TRINITY_DN1	2.98	−1.06	0.98	
16	TRINITY_DN7	2.89	−0.48	0.96	
17	TRINITY_DN3	2.65	−0.56	0.96	
18	TRINITY_DN1	2.6	−0.83	0.98	
19	TRINITY_DN5	2.52	−1.4	0.83	
20	TRINITY_DN5	2.4	−1.02	0.95	

Group Overexpression Sp

Spot Summary: B

metagenes = 12
genes = 63

<r> metagenes = 1
<r> genes = 0.76
beta: r2= 7.4 / log p= −Inf

samples with spot = 8 (33.3 %)
Zhewan−1(10) : 4 (100 %)
Zhongwan−6(10) : 4 (100 %)

Overview Map

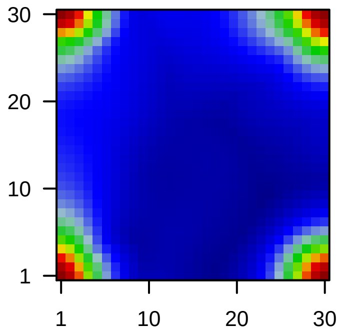

Spot

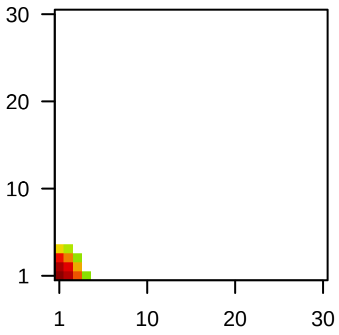

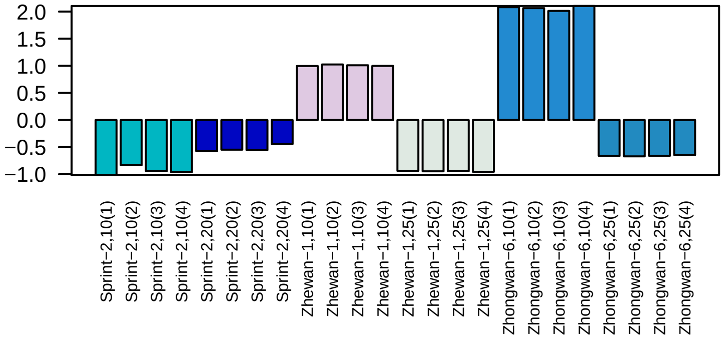

Spot Genelist

Rank	ID	max e	min e	r	Symbol	Description
1	TRINITY_DN1	13.12	−4.78	0.72		
2	TRINITY_DN7	11.07	−3.03	0.96		
3	TRINITY_DN1	8.39	−3.05	0.73		
4	TRINITY_DN1	6.17	−3.24	0.93		
5	TRINITY_DN4	5.77	−4.28	0.74		
6	TRINITY_DN3	5.76	−2.29	0.92		
7	TRINITY_DN1	5.25	−2.61	0.94		
8	TRINITY_DN1	4.57	−1.48	0.98		
9	TRINITY_DN2	4.53	−1.15	0.86		
10	TRINITY_DN3	4.41	−2.6	0.96		
11	TRINITY_DN2	4.05	−1.09	0.81		
12	TRINITY_DN3	3.87	−6.3	0.72		
13	TRINITY_DN3	3.49	−1.03	0.94		
14	TRINITY_DN7	3.48	−1.29	0.99		
15	TRINITY_DN2	3.3	−0.56	0.81		
16	TRINITY_DN2	3.29	−0.85	0.51		
17	TRINITY_DN1	3.26	−3.12	0.72		
18	TRINITY_DN1	2.87	−1.41	0.57		
19	TRINITY_DN2	2.8	−1.04	0.95		
20	TRINITY_DN8	2.78	−0.81	0.9		

Group Overexpression Sp

Spot Summary: C

metagenes = 11
genes = 58

<r> metagenes = 1
<r> genes = 0.62
beta: r2= 2.23 / log p= −Inf

samples with spot = 4 (16.7 %)
Sprint−2(20) : 4 (100 %)

Overview Map

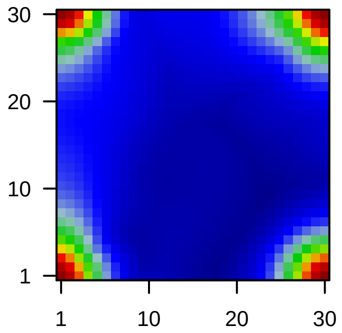

Spot

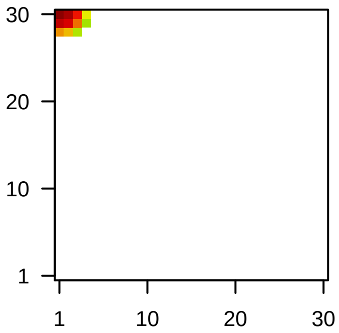

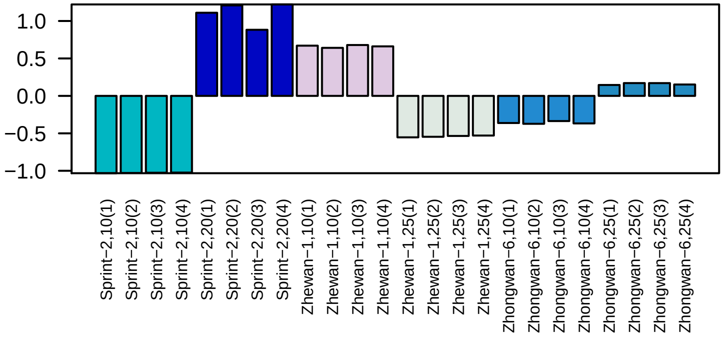

Spot Genelist

Rank	ID	max e	min e	r	Symbol	Description
1	TRINITY_DN4	8.82	−8.66	0.93		
2	TRINITY_DN4	8.39	−5.76	0.92		
3	TRINITY_DN4	5.37	−4.32	0.96		
4	TRINITY_DN1	3.77	−2.56	0.83		
5	TRINITY_DN3	3.65	−1.82	0.76		
6	TRINITY_DN5	3.42	−2.06	0.88		
7	TRINITY_DN4	2.8	−1.11	0.75		
8	TRINITY_DN2	2.61	−1.75	0.82		
9	TRINITY_DN5	2.36	−2.06	0.82		
10	TRINITY_DN5	2.24	−1.8	0.85		
11	TRINITY_DN4	2.18	−1.82	0.82		
12	TRINITY_DN2	2.08	−1.08	0.89		
13	TRINITY_DN1	2.07	−0.94	0.72		
14	TRINITY_DN4	1.85	−1.11	0.71		
15	TRINITY_DN5	1.76	−0.46	0.72		
16	TRINITY_DN1	1.73	−1.06	0.88		
17	TRINITY_DN1	1.71	−1.55	0.68		
18	TRINITY_DN5	1.6	−1.18	0.59		
19	TRINITY_DN9	1.54	−0.51	0.45		
20	TRINITY_DN1	1.54	−0.9	0.98		

Group Overexpression Sp[illegible]

Spot Summary: D

metagenes = 13
genes = 69

<r> metagenes = 1
<r> genes = 0.66
beta: r2= 3.95 / log p= −Inf

samples with spot = 8 (33.3 %)
Zhewan−1(25) : 4 (100 %)
Zhongwan−6(25) : 4 (100 %)

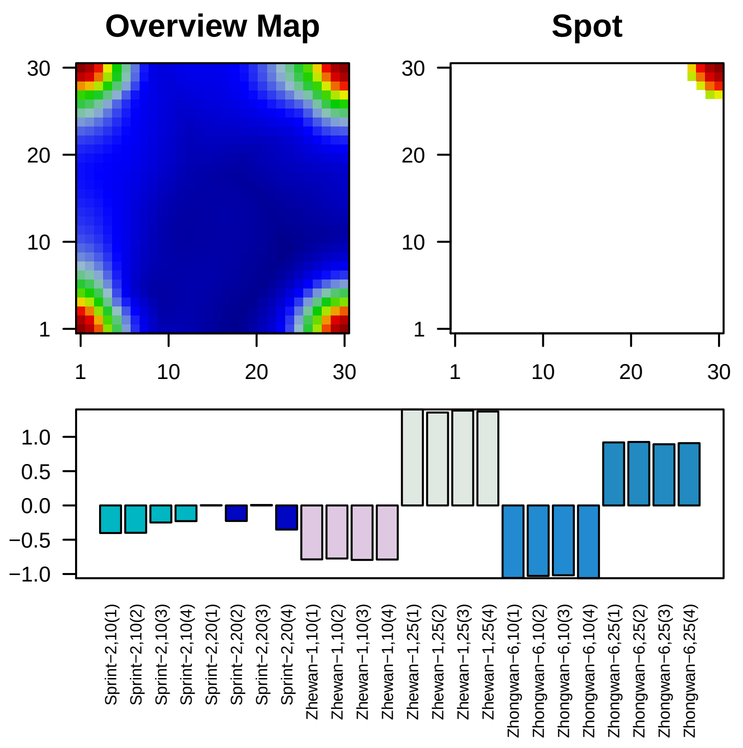

Spot Genelist

Rank	ID	max e	min e	r	Symbol	Description
1	TRINITY_DN1	10.4	−3.02	0.82		
2	TRINITY_DN4	7.91	−7.93	0.94		
3	TRINITY_DN4	7.74	−4.91	0.79		
4	TRINITY_DN1	7.72	−3.91	0.78		
5	TRINITY_DN2	5.9	−1.92	0.76		
6	TRINITY_DN2	4.81	−3.98	0.96		
7	TRINITY_DN5	4.22	−2.39	0.98		
8	TRINITY_DN2	4.11	−3.63	0.89		
9	TRINITY_DN3	3.31	−3.6	0.55		
10	TRINITY_DN1	3.23	−1.54	0.92		
11	TRINITY_DN8	2.99	−2.22	0.84		
12	TRINITY_DN7	2.62	−3.66	0.56		
13	TRINITY_DN6	2.53	−0.84	0.87		
14	TRINITY_DN1	2.27	−1	0.94		
15	TRINITY_DN2	2.25	−1.48	0.58		
16	TRINITY_DN4	2.14	−0.47	0.58		
17	TRINITY_DN1	2.09	−1.85	0.75		
18	TRINITY_DN9	2.04	−0.73	0.83		
19	TRINITY_DN9	1.98	−1.08	0.88		
20	TRINITY_DN4	1.92	−4.32	0.65		
